# Exosomes Derived from TIMP2-Modified Human Umbilical Cord Mesenchymal Stem Cells Enhance the Repair Effect in Rat Model with Myocardial Infarction Possibly by the Akt/Sfrp2 Pathway

**DOI:** 10.1155/2019/1958941

**Published:** 2019-04-28

**Authors:** Jing Ni, Xijun Liu, Yiheng Yin, Peiyu Zhang, Ya-Wei Xu, Zheng Liu

**Affiliations:** ^1^Department of Cardiology, Shanghai Tenth People's Hospital, Shanghai, China; ^2^Pan-Vascular Research Institute, Heart, Lung, and Blood Center, Tongji University School of Medicine, Shanghai, China

## Abstract

Exosomes derived from human umbilical cord mesenchymal stem cells (hucMSCs) are a promising new therapeutic option for myocardial infarction (MI). The tissue matrix metalloproteinase inhibitor 2, also known as TIMP2, is a member of the tissue inhibitor family of metalloproteinases. Since TIMP2-mediated inhibition of matrix metalloproteinases (MMPs) is a key determinant of post-MI remodeling, we analyzed the therapeutic effects of exosomes derived from TIMP2-overexpressing hucMSCs (huc-exo^TIMP2^) on the MI rat model. The huc-exo^TIMP2^ significantly improved *in vivo* cardiac function as measured by echocardiography and promoted angiogenesis in MI injury. It also restricted extracellular matrix (ECM) remodeling, as indicated by the reduced collagen deposition. In addition, huc-exo^TIMP2^ administration increased the *in situ* expression of the antiapoptotic Bcl-2 and decreased that of the proapoptotic Bax and pro-caspase-9 in the infracted myocardium. Meanwhile, huc-exo^TIMP2^ upregulated superoxide dismutase (SOD) as well as glutathione (GSH) and decreased the malondialdehyde (MDA) level in MI models. *In vitro* huc-exo^TIMP2^ pretreatment could inhibit H_2_O_2_-mediated H9C2-cardiomyocyte apoptosis and promote human umbilical vein endothelial cell (HUVEC) proliferation, migration, and tube formation, as well as decrease TGF*β*-induced MMP2, MMP9, and *α*-SMA secretion by cardiac fibroblasts (CFs). Besides that, huc-exo^TIMP2^ pretreatment also increased the expression of Akt phosphorylation in the infarcted myocardium, which may relate to a high level of secreted frizzled-related protein 2 (Sfrp2) in huc-exo^TIMP2^, indicating a mechanistic basis of its action. Importantly, Sfrp2 knockdown in huc-exo^TIMP2^ abrogated the protective effects. Taken together, huc-exo^TIMP2^ improved cardiac function by alleviating MI-induced oxidative stress and ECM remodeling, partly via the Akt/Sfrp2 pathway.

## 1. Introduction

Myocardial infarction (MI) results in the loss of cardiomyocytes and adverse remodeling of the extracellular matrix (ECM). The major mechanism underlying cardiomyocyte loss in the infarcted myocardium is oxidative stress [1], due to excessive reactive oxygen species (ROS) production, which impairs cellular functions and viability [2]. Therefore, improving cardiomyocyte survival and inhibiting apoptosis may be crucial for improving the prognosis of ischemic disease. In addition, the cardiac fibroblasts could be activated by myocardial injury triggering adverse ECM remodeling and excessive fibrosis. The ECM primarily composes of the type I and the type III collagen, which are crucial to maintain the normal cardiac structure, myocardial force, and myocardial systolic and diastolic coordination. Despite a plethora of treatments for MI, restoration of the scarred myocardial tissue is limited due to the nonrenewable nature of the myocardial cells [3].

Stem cell-based tissue regeneration is a promising therapeutic alternative for MI [4]. Transplantation of stem cells in the acute phase of MI inhibits apoptosis of cardiomyocytes, promotes neoangiogenesis, and reduces the local inflammatory response [5, 6]. The stem cells usually originate from the bone marrow and the fat autograft, as well as the umbilical cord. The human umbilical cord mesenchymal stem cells (hucMSCs) are highly suitable for the treatment of MI due to their high self-renewal ability, low immunogenicity, and fewer ethical issues regarding procurement [7, 8]. Studies have been increasingly focusing on paracrine actions of stem cells in recent years. Ma et al. found that Akt-modified hucMSCs secrete microparticles with a hydrodynamic radii ranging from 50 to 200 nm, which enhanced angiogenesis after MI injury [9]. Timmers et al. showed that the conditioned medium from human embryonic MSCs had the ability to repair myocardial ischemia-reperfusion injury, and the exosomes released from MSCs were the major regenerative factors [10]. Exosomes are cell-derived vesicles that transport proteins, lipids, DNA, and RNA and are involved in signal transduction, immune responses, antigen presentation, and would healing among other cellular processes [11], indicating their potential in bioactive therapies. In fact, hucMSC-derived exosomes have been shown to exert cardioprotective effects [12, 13].

The matrix metalloproteinases (MMPs), a group of proteolytic enzymes, could degrade the ECM and initiate the process of ventricular remodeling [14]. The tissue matrix metalloproteinase inhibitors (TIMPs) regulate MMP activity and maintain the balance between ECM breakdown and synthesis [15]. TIMP2, a member of the TIMP family, regulates the proteolytic activity of all MMPs and is involved in cell differentiation, growth, migration, angiogenesis, and apoptosis [16, 17]. TIMP2 knockout resulted in constitutive MMP9 activation abundantly and adverse MI remodeling *in vivo* [18]. Li et al. showed that TIMP2 and TIMP1 were increased by an endogenous miR-17 inhibitor, decreased MMP9 activity and infarct size, and improved cardiac function [19]. In fact, an aberrant MMP/TIMP ratio is the key factor driving ventricular remodeling in various cardiovascular diseases [20]. Valacca et al. demonstrated that TIMP2 binding to membrane-type 1 matrix metalloproteinase- (MT1-) MMP protected tumor cells against starvation-induced apoptosis by the regulation of the ERK1/2 and Akt signaling pathway [21]. The secreted frizzled- (Fz-) related protein 2 (Sfrp2), a downstream target of the PI3K/Akt signal pathway, regulates mesenchymal stem cell (MSC) survival [22]. In a recent study, Mastri et al. found an obvious improvement in cardiac function by injecting failing hamster hearts with the antibody targeted against the antifibrotic regulator Sfrp2 [23]. However, a potential involvement of the Akt/Sfrp2 axis in MI, or cardiac regeneration, is not completely clear.

In the present study, MI injury and H_2_O_2_ were used to induce cardiomyocyte injury with or without huc-exo^TIMP2^ administration, respectively, *in vivo* and *in vitro*. We confirmed that MI could induce cardiomyocyte apoptosis as well as ECM remodeling and huc-exo^TIMP2^ partially abolished such effect. In addition, we also found that the Akt/Sfrp2 signal pathway may exert the antiapoptotic action on cardiomyocytes partly by inhibiting oxidative stress.

## 2. Materials and Methods

### 2.1. Ethics Statement

All the animal procedures were conducted in agreement with the Guidelines of Shanghai Laboratory Animal Center and the Policies on the Use of Humans and Animals in Research of the Shanghai Tenth People's Hospital (SYXK: 2014-0026) and confirmed to the principles outlined in the Declaration of Helsinki. The experimental animals were treated humanely, and all efforts were made to ease their discomfort.

### 2.2. Culture and Identification of hucMSCs

The hucMSCs were purchased from Shycbio (Shanghai, China) and seeded into Dulbecco's modified Eagle's medium F12 (DMEM/F12) medium containing 10% fetal bovine serum (FBS) and 1% PS (all from Gibco, Thermo Fisher Scientific, Waltham, MA, US) at 37°C with 5% CO_2_. The culture medium was changed every 72 hrs. When the adherent reached 80% confluency, they were harvested and treated with 0.05% trypsin/0.5 mM EDTA (Sigma-Aldrich, St. Louis, MO, US), washed with flow cytometry buffer (PBS containing 0.5% FBS and 0.02% sodium azide), and divided into aliquots of 0.5-1 × 10^6^ cells. The cells were cultured with various fluorescein isothiocyanate- (FITC-) conjugated or phycoerythrin- (PE-) conjugated monoclonal antibodies (Santa Cruz Biotechnology, Dallas, TX, US), including CD90, CD73, CD105 and CD45, and for 30 min at 37°C. The labeled cells were detected by a FACScan flow cytometer (Becton Dickinson, Franklin Lakes, NJ, US), and nonspecific IgG-labeled cells were used as controls.

### 2.3. Osteogenic and Adipogenic Differentiation

The hucMSCs from passage 2 (1-2 × 10^4^ cells/per well) were plated on 24-well plates and cultured in DMEM/F12 containing 10% FBS, 0.1 mM dexamethasone, 10 mM *β*-glycerophosphate, 50 mg/L ascorbic acid, and 4 mg/ml bFGF for osteogenic differentiation (all from Sigma-Aldrich, St. Louis, MO, US). The medium was replaced every 72 hrs, and 3 weeks later, calcium deposition was detected by alizarin red staining.

For adipogenic differentiation, cells of the 2nd passage were seeded in a 24-well plate in DMEM/F12 containing 10% FBS, 1 mM dexamethasone, and 10 mg/ml insulin (all from Sigma-Aldrich). 2 weeks later, Oil Red O staining was used to measure lipid vesicles. The pictures were captured by an Olympus IX51 light microscope (Olympus Canada Inc., Ontario, Canada).

### 2.4. Transduction of hucMSCs with Lentivirus-TIMP2

hucMSCs were transduced with the lentiviral-TIMP2 (MOI = 80) or lentiviral-NC (empty vector, MOI = 80) using 8 *μ*g/ml polybrene at 37°C in 1.0 ml serum-free medium for 24 hrs (BioLink, Shanghai, China). 48 hrs after infection, the positively transduced cells showed green fluorescence. The expression level of TIMP2 in the transfected hucMSCs (hucMSCs^TIMP2^) was detected by using qPCR and Western blotting.

### 2.5. Harvest and Identification of Exosomes from Lentivirus-hucMSCs

Exosomes were purified from TIMP2-transduced hucMSC- (huc-exo^TIMP2^-) conditioned media by using ultracentrifugation (Beckman Coulter, Brea, CA, US). Briefly, the hucMSCs^TIMP2^ were cultured till 80% confluency, washed with PBS, and reseeded in DMEM/F12 (HyClone, GE Healthcare, Waukesha, WI, US) including 10% exosome-free FBS (centrifuged at 100,000g for 8 hrs to eliminate preexisting bovine-derived exosomes) and 1% PS. After 24 hrs, the conditioned medium was centrifuged for 10 min at 2,000g to remove cell debris. The huc-exo^TIMP2^ were purified from the supernatant by ultracentrifugation at 100,000 g for 70 min. All procedures were carried out at 4°C. Exosomes were finally filtered through 0.22 *μ*m Whatman polycarbonate filters (Whatman, Maidstone, UK) and stored at -80°C. hucMSCs with lentivirus-NC were subjected to the same protocol to obtain the huc-exo^NC^.

The Hitachi H-7650 transmission electron microscope (Hitachi, Tokyo, Japan) was used to observe the exosomal morphology of both huc-exo^NC^ and huc-exo^TIMP2^ directly. The BCA protein assay kit (Beyotime, Shanghai, China) was used to measure the protein concentration of exosomes and characterized by Western blot with anti-CD9 and anti-CD63 antibodies (all from Abcam, Cambridge, MA, US).

### 2.6. Cell Isolation and Culture

Neonatal cardiomyocytes were isolated from Sprague Dawley rats (3 days old) by multiple rounds of digestion using 1% collagenase II (Yishen, Shanghai, China) for 60 min. The digested cells were filtered through a 100 *μ*m mesh (Whatman, Maidstone, UK), centrifuged at 1,000 rpm for 10 min, and resuspended in DMEM containing 20% FBS and 1% PS. After a 45 min incubation, the neonatal cardiomyocytes in suspension were aspirated carefully from the adherent fibroblasts, reseeding in a new culture flask and cultured for another 48 hrs. The media were replaced every three days.

H9C2 cells were seeded in high glucose DMEM and supplemented with 10% FBS and 1% PS. Human umbilical cord vein endothelial cells (HUVECs) were cultured in a complete endothelial cell growth medium (ATCC, Manassas, VA, US).

The cells were treated as follows: (i) H_2_O_2_ (100 *μ*M for 24 hrs), (ii) TGF*β* (10 ng/ml for 24 hrs), (iii) huc-exo^NC^ (50 *μ*g/ml for 6 hrs) followed by H_2_O_2_ for 24 hrs, (iv) huc-exo^NC^ (50 *μ*g/ml for 6 hrs) followed by TGF*β* for 24 hrs, (v) huc-exo^TIMP2^ (50 *μ*g/ml for 6 hrs) followed by H_2_O_2_ for 24 hrs, and (vi) huc-exo^TIMP2^ (50 *μ*g/ml for 6 hrs) followed TGF*β* for 24 hrs. Untreated controls were also included. Following the different treatments, the medium was replaced with serum-free medium and cells were incubated for another 24 hrs.

### 2.7. PKH67 Labeling and Fluorescent Microscopy

The exosomes (huc-exo^NC^ or huc-exo^TIMP2^) were cultured with 1 *μ*g/ml PKH67 dye (Invitrogen, Waltham, MA, US) for 30 min in the dark, washed with PBS, and centrifugated at 100,000g for 1.5 hrs to remove the unbound dye. H9C2 cardiomyocytes and cardiac fibroblasts were cultured with 50 *μ*g/ml PKH67-labeled exosomes for 1 hr, 6 hrs, 12 hrs, and 24 hrs. The cells were washed by PBS and fixed with 4% paraformaldehyde for 10 min. After counterstaining with 1 mg/ml 4′,6-diamidino-2-phenylindole (DAPI, Yishen, Shanghai, China) for 5 min, the cells were observed under a Leica DMI6000B fluorescent microscope (Leica Microsystems Ltd., Wetzlar, Germany).

### 2.8. Cell Viability Assay

The CCK-8 kit (Dojindo Molecular Technologies Inc., Rockville, MD, US) was used to test cell viability. Briefly, cells were seeded in 96-well plates (1 × 10^4^ cells/per well) in 100 *μ*l DMEM containing 10% FBS overnight. After pretreatment with different concentrations of huc-exo^NC^ or huc-exo^TIMP2^ (0.1, 1, 10, and 50 *μ*g/ml) for 24 hrs, 10 *μ*l CCK8 solution was added to each well and incubated at 37°C for 1 hr. The absorbance was measured at 450 nm using an enzyme-linked immunosorbent assay plate reader (Bio-Rad Laboratories, Hercules, CA, US).

### 2.9. *In Vivo* Myocardial Infarction Model

Male Sprague Dawley rats (6 weeks old, weighting 130-180 g) were procured from the Shanghai Laboratory Animal Center (Shanghai, China) and housed under standard laboratory conditions for 1 week with free access to food and water. The rats were randomized into four groups: (i) the blank control (*n* = 6), (ii) the sham group (MI with PBS, *n* = 6), (iii) MI with the huc-exo^NC^ transplantation group (*n* = 8), and (iv) MI with the huc-exo^TIMP2^ transplantation group (*n* = 8). To induce MI, the rats were anesthetized with pentobarbital (60 mg/kg i.p., Shenggong, Shanghai, China) and ventilated using a SCIREQ flexiVent small animal ventilator (SCIREQ, Montreal, Ontario, Canada) to maintain the body temperature at 37°C during the surgery. Anterior thoracotomy was performed to expose the hearts, and the proximal left descending coronary artery was ligated by a 6-0 silk suture. End-expiratory pressure was applied to fully inflate the lungs. After ligation, the PBS, huc-exo^NC^ (50 *μ*g/ml), and huc-exo^TIMP2^ (50 *μ*g/ml) were injected at three different sites in the peri-infarct zone with a 28-gauge syringe. Ad libitum feeding was resumed after the surgery. Echocardiography was performed after 30 days post-MI. After the last evaluation, the animals were decapitated and the whole hearts were excised and weighed. A portion of the ventricular tissue was preserved at -80°C for molecular analyses, and the remainder was used for histopathology and study of the infarcted area.

### 2.10. Echocardiography

Echocardiography was performed using a Vevo 770 system (VisualSonics Inc., Toronto, Canada) under anesthesia with inhalation of 1.5% isoflurane-O_2_. During the scan, the rats were positioned in a left lateral decubitus form, and left ventricular (LV) M-mode and two-dimensional images were obtained by parasternal short axis view at the papillary muscle level. The parasternal long axis views were obtained and recorded to ensure that visualization of the mitral and aortic valves and the apex was visualized. The transmission frequency was 10 MHz, the depth 2.5 cm, and the frame rate was 225-350 frames per second. The heart rate was recorded as the R-R interval of the electrocardiogram signal. The left ventricular end-systolic dimension (LVESd) and left ventricular end-diastolic volume (LVEDd) were measured from the M-mode tracing. The ejection fraction (EF%) was calculated as EF% = [(LVEDd^3^ − LVESd^3^)/LVDD^3^]∗100, and the shortening fraction (FS%) used the following equation: FS% = [(LVEDd − LVESd)/LVEDd]∗100. All measurements were made by a single observer blinded to the groups of experiments. An average of three consecutive measurements of each variable was used for further analysis.

### 2.11. Histopathological Examination of Heart Tissues

Isolated hearts were fixed with 4% paraformaldehyde and embedded in paraffin, and serial 4-5 *μ*m sections were prepared. The sections were stained with hematoxylin and eosin (H&E) for morphological analysis and with Masson's trichrome (MT) to detect the collagen deposition and smooth muscle thickness. The slides were observed under an Olympus IX51 light microscope (Olympus Canada Inc., Ontario, Canada), and stained cells were counted in 3 randomly selected fields by an investigator blinded to the sample.

### 2.12. Immunofluorescence Assay

The rats were deeply anesthetized and perfused with ice cold PBS/4% paraformaldehyde, following which the hearts were resected and immersed in 30% sucrose. The tissues were cut into 5 *μ*m sections using a freezing microtome. After blocking in 5% BSA for 1 hr, the sections were incubated overnight at 4°C with CD31 and *Lycopersicon esculentum* lectin antibodies (1 : 50, Abcam, Cambridge, MA, US). The slides were washed with PBS and then incubated with the fluorochrome-labeled goat anti-rabbit IgG conjugated-secondary antibody or goat anti-mouse IgG conjugated-secondary antibody (1 : 1000, Life Technologies, Grand Island, NY, US) for 1 hr at room temperature in the dark.

Cultured cardiac fibroblasts were fixed in 4% paraformaldehyde for 10 min, permeabilized with 0.2% Triton X-100 (Sigma-Aldrich) for 5 min, and blocked with normal goat serum (Boster, Wuhan, China) for 30 min at room temperature. The cells were incubated overnight at 4°C with the anti-*α*-SMA antibody (1 : 200, Cell Signaling Technology, Danvers, MA, US), followed by a fluorochrome-labeled goat anti-rabbit IgG conjugated-secondary antibody (1 : 1000, Life Technologies) for 1 hr at room temperature. After that, sections were counterstained with 4′,6-diamidino-2-phenylindole (DAPI, Boster Biological Technology, Pleasanton, CA, US). Inverted fluorescence microscopy (Leica Microsystems Ltd., Wetzlar, Germany) was used to capture fluorescent images.

### 2.13. Measurement of Antioxidant Enzymes and Intracellular Reactive Species Generation

The malondialdehyde (MDA) content, superoxide dismutase (SOD) activity, and glutathione (GSH) concentration were measured in rat heart tissues using assay kits purchased from Jiancheng Bioengineering Institute (Nanjing, China) following the manufacturer's instructions [24].

To measure the oxidative level, flow cytometry was used to monitor the intracellular ROS. In brief, the cells were seeded on 96-well plates, collected, and incubated with 2′,7′-dichlorofluorescein-diacetate (DCFH-DA, 1 *μ*M) dye in the dark for 30 min at 37°C. Then, the cells were washed with PBS, and the fluorescent intensity of intracellular ROS was detected by the FACSAria III flow cytometer (Becton Dickinson, Franklin Lakes, NJ, US) with the excitation wavelength at 488 nm and emission wavelengths at 525 nm.

### 2.14. TUNEL Staining

Terminal deoxynucleotidyl transferase- (TdT-) mediated deoxyuridine triphosphate (dUTP) nick end labeling (TUNEL) was performed using an *in situ* apoptosis detection kit (Beyotime, Jiangsu, China). Briefly, the heart sections were deparaffinized with xylene, rehydrated with ethanol, and rinsed twice in 0.1 M Tris-HCl buffer (pH 7.4) and then twice with PBS. After blocking the sections with 0.3% H_2_O_2_ for 10 min at room temperature, TdT and dUTP reactions were performed for 1 hr at 37°C. In addition, the TUNEL assay was performed to analyze the cell apoptosis. Briefly, the cells were fixed in 4% paraformaldehyde and permeabilized in 0.03% Triton X-100. Then, the cells were incubated in TUNEL reaction mixture for 1 hr at 37°C. For the negative control, the TdT enzyme was omitted. Nuclei were counterstained with DAPI, and apoptosis was calculated as the number of TUNEL-positive cells in each group and captured with a Leica (Leica Microsystems Ltd., Wetzlar, Germany) fluorescence microscope.

### 2.15. Flow Cytometric Analysis of Apoptosis

Early apoptotic cells were detected using an annexin V-fluorescein isothiocyanate (FITC)/propidium iodide (PI) detection kit (Becton Dickinson, Franklin Lakes, NJ, US). Briefly, cells (1 × 10^5^ cells/well) were harvested from each group and added to 500 *μ*l annexin binding buffer. Subsequently, cells were stained with 5 *μ*l annexin V reagent and 10 *μ*l PI in Ca^2+^ enriched binding buffer for 15 min at 37°C in the dark. Then, cells were analyzed by the FACSAria III flow cytometer (Becton Dickinson), and at least 1 × 10^4^ cells were acquired per sample.

### 2.16. Tube Formation Assay

The formation of tube-like structures by the HUVECs was detected *in vivo* on ECM gel (Sigma-Aldrich). Briefly, the HUVECs were seeded onto ECM-coated (150 *μ*l/well) plates at the density of 1 × 10^4^ cells/well and treated with exosomes (50 *μ*g/ml) in the presence/absence of 100 *μ*M H_2_O_2_ for 12 hrs. The endothelial network formation was observed and captured under an Olympus IX51 microscope. The numbers and length of the tube structures were counted in three randomly selected fields per well using ImageJ software ver. 1.52.

### 2.17. Wound Healing Assay

HUVECs were seeded in 6-well plates and cultured till 90% confluency. After switching to serum-free media, the monolayer was scratched with a sterile 200 *μ*l pipette tip, and migration of the cells to the “wound” area was monitored at 0 hr and 12 hrs under an inverted microscope (Olympus IX51, Ontario, Canada). Cell migration was analyzed using ImageJ software ver. 1.52. The experiment was conducted in triplicate.

### 2.18. RNA Extraction and Real-Time Polymerase Chain Reaction (RT-PCR)

Freshly isolated heart tissues were washed with RNase-free water, and total RNA was isolated using TRIzol reagent (Life Technologies) according to the manufacturer's instructions. PrimeScript™ RT Reagent Kit (Takara Bio Inc., Shiga, Japan) was used to reverse transcribe cDNAs. The PCR was performed in an Applied Biosystems 7500 Real-Time PCR System (Applied Biosystems, Life Technologies) using an SYBR® Premix Ex Taq™ Reagent Kit (Takara). The reaction conditions were set as follows: 95°C for 10 s, 40 cycles of 95°C for 5 s and 60°C for 31 s, one cycle of 95°C for 15 s, 60°C for 30 s, and 95°C for 15 s. The data were normalized to the internal control GAPDH and analyzed using the 2-ΔΔCT method. Primer sequences used for this study were as follows: MMP2: forward, 5′-ACGCTGATGGCGAGTACTGCA-3′, reverse, 5′-CCATGGTAAACAAGGCTTCGTG-3′; MMP9: forward, 5-CCTCTGCATGAAGACGACATAA-3′, reverse, 5′-GGTCAGGTTTAGAGCCACGA-3′; TIMP2: forward, 5′-TATTGTGCCCTGGGACACG-3′, reverse, 5′-GTCCATCCAGAGGCACTCATC-3′; and GAPDH: forward, 5′-AAACTCACTGGCATGGCCTT-3′, reverse, 5′-TTAGCAGCTTTCTCCAGGCG-3′.

### 2.19. shRNA Transfection

The target sequence for Sfrp2-specific short hairpin RNA (shRNA, Linker, Shanghai, China) was designed as follows: 5′-GATCCGGAAGCTCCAAAGGTATGTGAATCAAGAGTTCACATACCTTTGGAGCTTCTTTTTTGG-3′. The shRNA-Sfrp2 (sh-Sfrp2) were transfected into the pLKO.1 lentiviral vector. The hucMSCs^TIMP2^ were seeded into six-well plates and transfected with the lentivirus using polybrene (10 *μ*g/ml, Sigma-Aldrich). After 6 hrs, the lentiviral media was replaced with the completed culture media for another 48 hrs. An empty vector which only expressed green fluorescent protein (GFP) was used as the negative control (sh-NC). Then, cells were harvested at the indicated time points and detected the infection efficiency by RT-PCR.

### 2.20. Western Blotting

Cells were harvested and lysed in RIPA buffer (Beyotime, Shanghai, China), and the lysates containing 50 *μ*g protein were boiled for 10 min at 100°C. The samples were loaded and separated in 10%-15% SDS-PAGE gel and then transferred onto polyvinylidene fluoride membranes (Millipore, Billerica, MA, US). The blots were blocked with 5% nonfat dry milk in PBST for 1 hr at room temperature and then incubated overnight at 4°C with primary antibodies against MMP2, MMP9, Bcl2, Bax, pro-caspase-9, TIMP2, phosphorylate Akt, or total Akt (1 : 1000, Santa Cruz Biotechnology) and CD9, CD63, *α*-SMA, or Sfrp2 (1 : 800, Cell Signaling Technology). After washing with PBST, the blots were incubated with horseradish peroxide goat anti-rabbit secondary antibody or horseradish peroxide goat anti-rat secondary antibody (1 : 10000; Yishen, Shanghai, China) for 1 hr at room temperature. GAPDH was also detected as an internal control (1 : 1000, Millipore). Positive bands were visualized by using the enhanced chemiluminescence kit (Amersham, GE Healthcare, Waukesha, WI, US) and quantified by ImageJ software ver. 1.52.

### 2.21. Statistics

All the experiments were performed in triplicate unless otherwise indicated. All the data are expressed as meanvalue ± standard error of mean (SEM) and compared by one-way ANOVA using the GraphPad Prism 6.0 software (GraphPad Software, San Diego, CA, US), followed by the Bonferroni multiple comparison test. *p* values < 0.05 are considered statistically significant.

## 3. Results

### 3.1. Characterization of hucMSCs and hucMSC-Derived Exosomes

**Figure 1:**
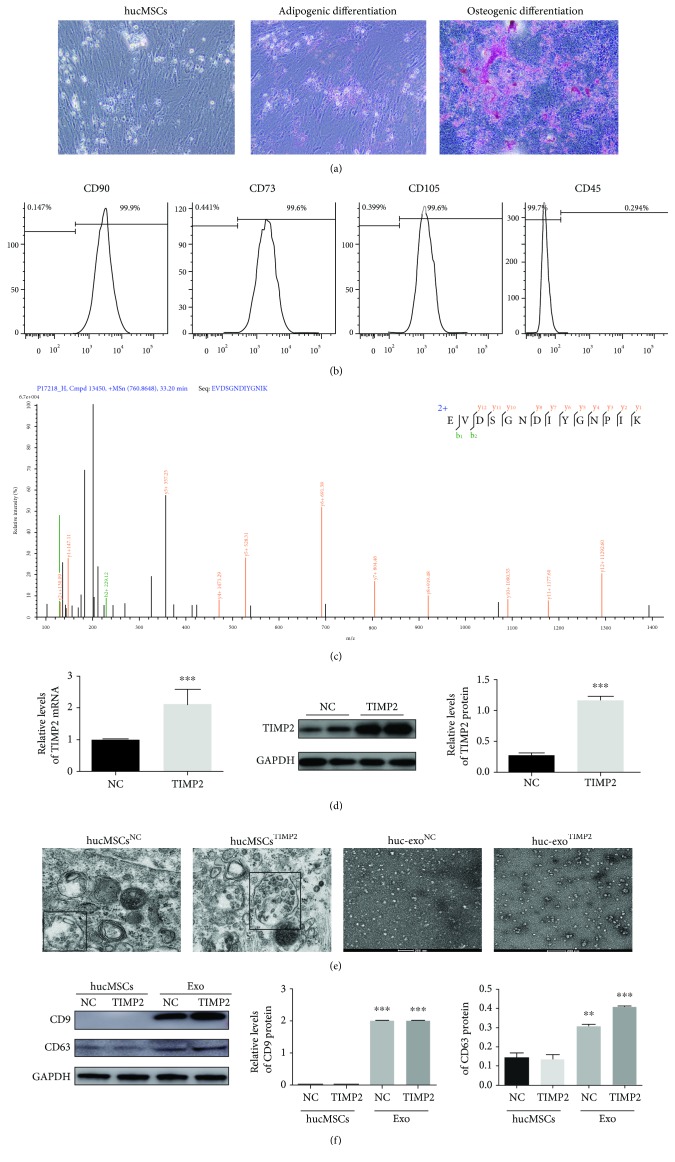
Characterization of hucMSCs and hucMSC-derived exosomes. (a) Representative images of spindle-shaped hucMSCs and terminally differentiated adipogenic (Oil Red O-stained) and osteogenic (alizarin A-stained) cells. Scale bar: 50 *μ*m. (b) Flow cytometric plots showing enriched CD90+, CD73+, CD105+, and CD45- hucMSCs. (c) Mass spectrometry results of the TIMP2 level in hucMSC-derived exosomes. (d) Quantification of TIMP2 mRNA and protein expression in TIMP2-transfected hucMSCs. (e) TEM micrographs showing the morphology of hucMSC^NC^- and hucMSC^TIMP2^-derived exosomes. Scale bar: 500 nm/200 nm. (f) Immunoblots showing the expression levels of CD9 and CD63 in hucMSCs^NC^, hucMSCs^TIMP2^, huc-exo^NC^, and huc-exo^TIMP2^. ^∗^*p* < 0.05, ^∗∗^*p* < 0.01, and ^∗∗∗^*p* < 0.001.

hucMSCs cultured *in vitro* showed the characteristic spindle-shaped morphology and were able to differentiate into adipocytes and osteocytes in response to appropriate inducement (Figure 1(a)). To gain further understanding of hucMSCs, the immunophenotype of the hucMSCs from the third passage was positive for CD90, CD73, and CD105 and negative for CD45, indicating an enriched population of hucMSCs (Figure 1(b)).

Mass spectrometry analysis showed high endogenous levels of the TIMP2 protein in the hucMSCs-exo (Figure 1(c)), and overexpression of the exogenous TIMP2 in hucMSCs was validated by qPCR and Western blotting (Figure 1(d)). TIMP2 mRNA and the protein level were significantly increased in hucMSCs^TIMP2^ than in hucMSCs^NC^.

The exosomes were characterized both morphologically and immunophenotypically. As shown in the TEM micrographs in Figure 1(e), numerous multivesicular bodies were present in the cytoplasm of hucMSCs^NC^ and hucMSCs^TIMP2^, in addition to the secreted membrane-bound, cup-shaped, exosome-like vesicles (40 to 90 nm in diameter). In addition, huc-exo^NC^ and huc-exo^TIMP2^ expressed the exosomal markers CD9 and CD63 in a higher extent than hucMSCs (Figure 1(f)). Collectively, these results detected that both hucMSCs^NC^ and hucMSCs^TIMP2^ secrete exosomes and that these exosomes secreted by these cell populations have similar biochemical characterization.

### 3.2. hucMSC-Derived Exosomes Could Be Internalized by Cardiomyocytes

**Figure 2:**
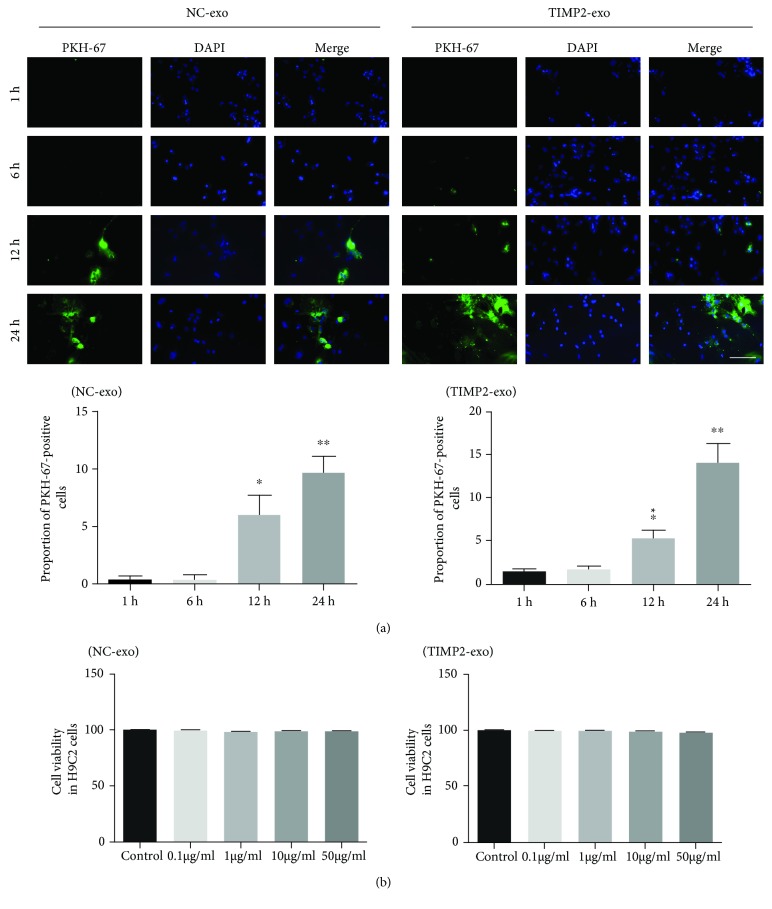
Exosome internalization and cell viability in H9C2 cells *in vitro*. (a) Representative fluorescent images of H9C2 cells incubated with PKH67-labeled huc-exo^NC^ and huc-exo^TIMP2^ (green) at 1 hr, 6 hrs, 12 hrs, and 24 hrs and quantitation of PKH67-positive cells. Scale bar: 50 *μ*m. (b) A percentage of viable H9C2 cells was cocultured with different concentrations of exosomes (0.1, 1, 10, and 50 *μ*g/ml) for 24 h and then analyzed with the CCK-8 kit. OD540: optical density at 540 nm. ^∗^*p* < 0.05 and ^∗∗^*p* < 0.01.

The internalization of the exosomes by H9C2 cardiomyocytes was tracked by labelling the huc-exo^NC^ and huc-exo^TIMP2^ with the fluorescent PKH67. As shown in Figure 2(a), both PKH67-labeled exosomes mainly localized in the cytoplasm of H9C2 cardiomyocytes in a time-independent manner.

In addition, cells cultured with different concentrations of the exosomes (0.1, 1, 10, and 50 *μ*g/ml) showed no changes in viability (Figure 2(b)), indicating that the hucMSC-derived exosomes had no toxic effects on the H9C2 cardiomyocytes.

### 3.3. huc-exo^TIMP2^ Alleviated Cardiac Remodeling and Dysfunction *In Vivo*

**Figure 3:**
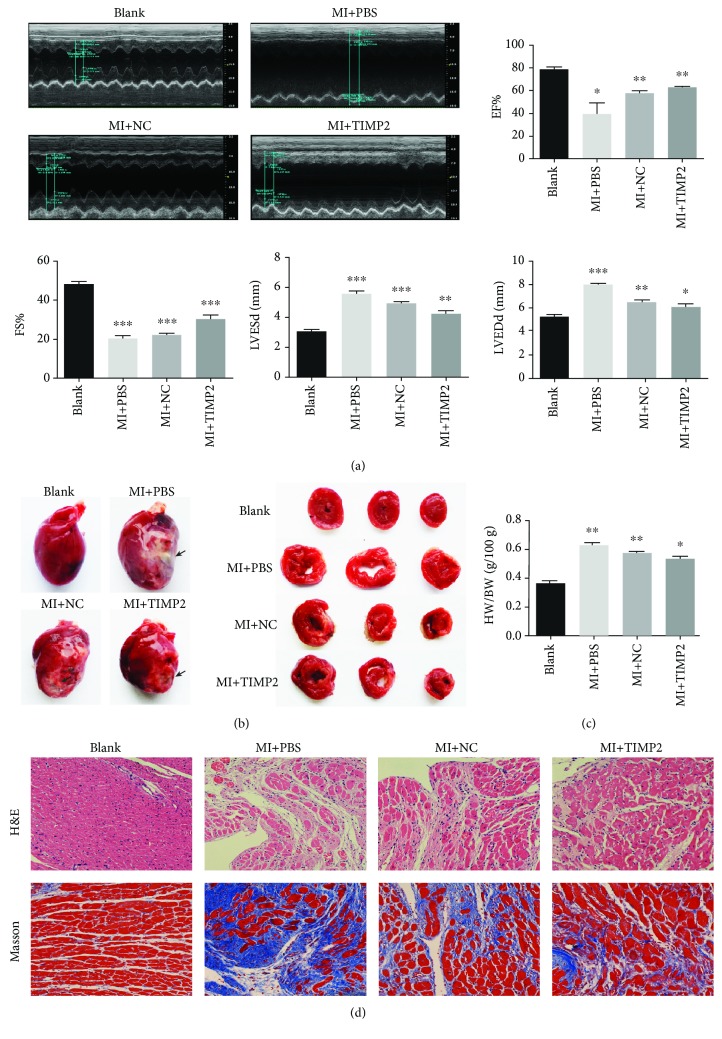
Effect of huc-exo^TIMP2^ on post-MI cardiac function. (a) EF%, FS%, LVESd, and LVEDd values at 30 days after injection of exosomes. (b) Representative pictures showing the macroscopic appearance of hearts at 30 days post-MI. Arrows indicate infarcted areas. (c) Heart weight/body weight (HW/BW) ratios in different groups. (d) Representative pictures of H&E and Masson trichrome-stained heart sections in different groups. Scale bar: 50 *μ*m. Blank: rats without surgery; MI+PBS: MI rats treated with PBS; NC: MI rats treated with huc-exo^NC^; TIMP2: MI rats treated with huc-exo^TIMP2^. ^∗^*p* < 0.05, ^∗∗^*p* < 0.01, and ^∗∗∗^*p* < 0.001.

To assess the potential cardioprotective effects of the hucMSC-derived exosomes, huc-exo^NC^ and huc-exo^TIMP2^ were injected directly into the MI rat myocardium. After 30 days, post-MI cardiac function was measured by echocardiography, and EF%, FS%, LVESd, and LVEDd were evaluated. Compared to the placebo (PBS) group, rats injected with huc-exo^TIMP2^ showed significantly increased LV contractility (EF%) and FS% post-MI. In addition, the D-value of LVESd and LVEDd in the huc-exo^TIMP2^ group was lower compared to other groups (Figure 3(a)).

Histologically, the infarct size in the huc-exo^TIMP2^ transplanted rats was significantly reduced compared to the huc-exo^NC^ and placebo groups (Figure 3(b)). Furthermore, huc-exo^TIMP2^ treatment significantly decreased ventricular dilation as measured in terms of heart weight/body weight ratio (Figure 3(c)), less myocardial remodeling and fibrosis as assessed by an H&E stain and Masson's trichrome stain (Figure 3(d)) compared with other groups. Taken together, huc-exo^TIMP2^ showed a potent cardioprotective action in infarcted rat hearts by reversing functional decompensation and pathological remodeling, as well as heart failure.

### 3.4. huc-exo^TIMP2^ Attenuated Oxidative Stress-Induced Apoptosis in MI Injury

**Figure 4:**
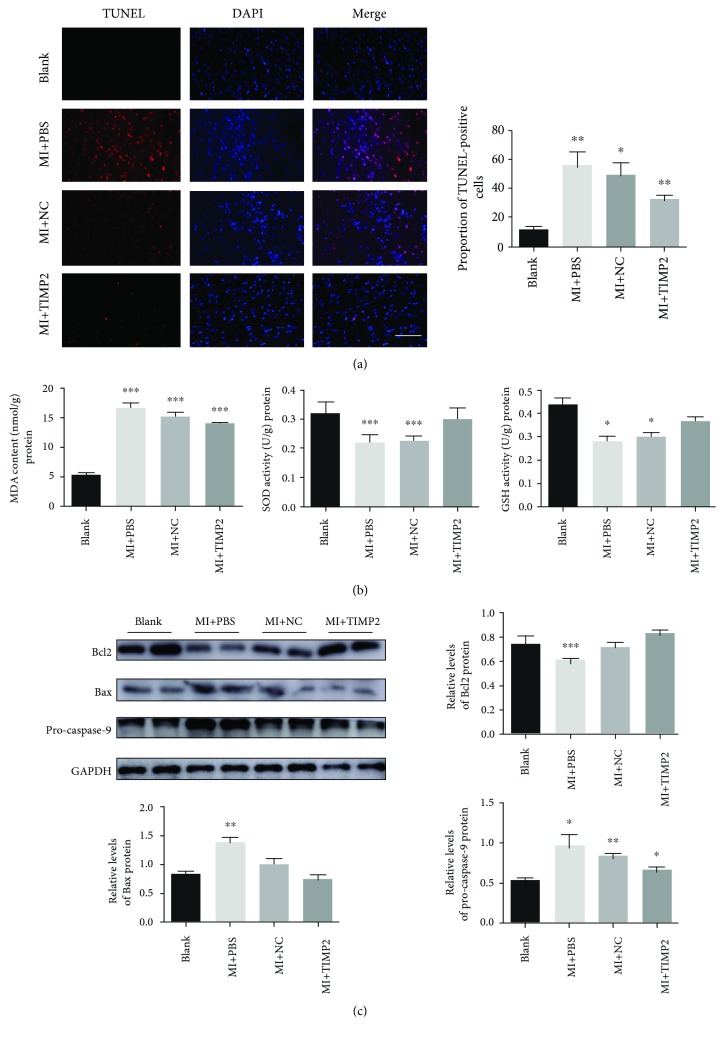
huc-exo^TIMP2^ ameliorated oxidative stress-induced cardiomyocyte apoptosis. (a) TUNEL^+^ nuclei in cardiac sections. Scale bar: 50 *μ*m. (b) The levels of MDA, SOD, and GSH concentrations in the heart tissues of different groups. (c) Immunoblots showing the expression levels of Bcl2, Bax, and pro-caspase-9 in heart tissues of different groups; comparison of quantified protein levels. Blank: rats without surgery; MI+PBS: MI rats treated with PBS; NC: MI rats treated with huc-exo^NC^; TIMP2: MI rats treated with huc-exo^TIMP2^. ^∗^*p* < 0.05, ^∗∗^*p* < 0.01, and ^∗∗∗^*p* < 0.001.

To confirm whether huc-exo^TIMP2^ was involved in oxidative stress-induced apoptosis, TUNEL staining was used. As shown in Figure 4(a), a significant decrease in TUNEL+ cells was detected in cardiac sections of huc-exo^TIMP2^ transplanted rats compared with the placebo groups. Besides that, the MDA concentration was significantly increased, and SOD and GSH activities were decreased in MI injury-induced hearts, while huc-exo^TIMP2^ administration could reverse this trend (Figure 4(b)). These above intracellular antioxidant factors are all involved in counteracting and clearing ROS [25]. To further investigate how huc-exo^TIMP2^ inhibited myocardium apoptosis, Western blot was used to determine the apoptosis-related protein expression. As shown in Figure 4(c), the Bcl2 level was significantly decreased, while Bax, as well as pro-caspase-9, was increased at 30 days after MI injury. Upregulation of TIMP2 in huc-exo could reverse the MI injury-induced Bcl2 expression and inhibit Bax and pro-caspase-9 levels. Collectively, these results showed that huc-exo^TIMP2^ plays an important role in inhibiting myocyte apoptosis and promoting the antioxidant defense system that in turn contribute to the cardiac repair process.

### 3.5. huc-exo^TIMP2^ Promoted Angiogenesis Both *In Vivo* and *In Vitro*

**Figure 5:**
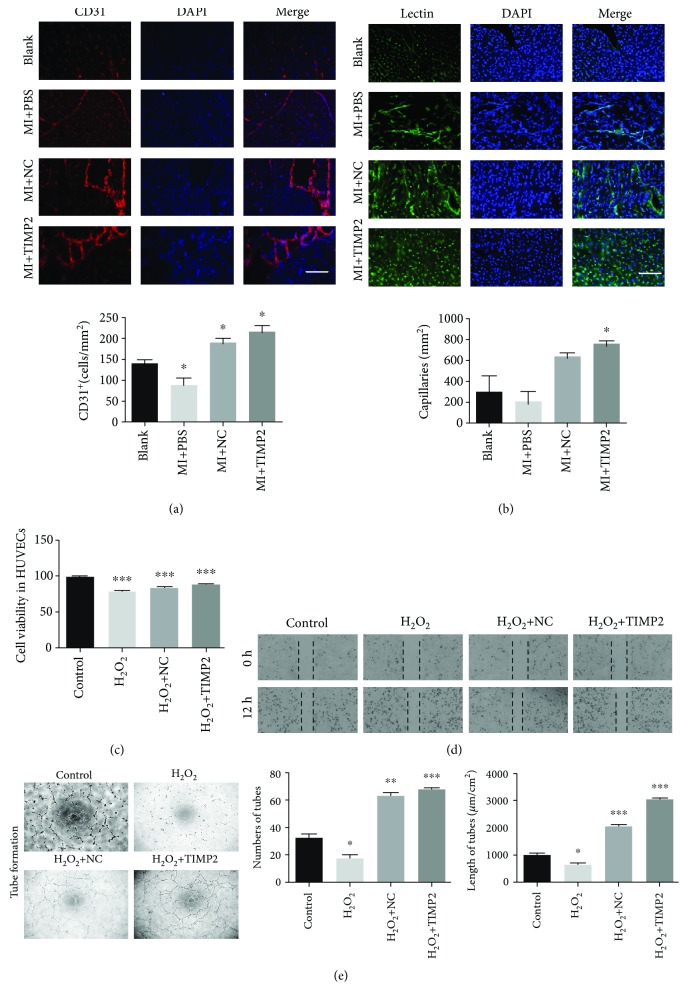
huc-exo^TIMP2^ promoted angiogenesis both *in vivo* and *in vitro*. (a) Representative images of heart sections showing *in situ* expression of CD31 and lectin; quantitative comparison of CD31 and lectin fluorescence intensity. CD31: red, lectin: green, DAPI: blue; scale bar: 50 *μ*m. Blank: rats without surgery; MI+PBS: MI rats treated with PBS; NC: MI rats treated with huc-exo^NC^; TIMP2: MI rats treated with huc-exo^TIMP2^. (b) Viability of differentially treated HUVECs. OD540: optical density at 540 nm. (c) Representative images of the wound healing assay showing *in vitro* migration of HUVECs. Scale bar: 50 *μ*m. (d) Representative images of *in vitro* tube formation by HUVECs; comparison of tube number and length among different groups. Scale bar: 50 *μ*m. Control: untreated; H_2_O_2_: treated with H_2_O_2_ alone; NC: treated with huc-exo^NC^ following H_2_O_2_; TIMP2: treated with huc-exo^TIMP2^ following H_2_O_2_. ^∗^*p* < 0.05, ^∗∗^*p* < 0.01, and ^∗∗∗^*p* < 0.001.

Angiogenesis is an important factor influencing ischemic myocardial repair. To determine the angiogenic activity in the differentially treated MI rats, their heart tissue sections were probed for CD31 and lectin. Compared to the placebo-treated MI rats, huc-exo^TIMP2^ treatment significantly increased the *in situ* expression levels of CD31 (Figure 5(a)) and lectin (Figure 5(b)).

In addition, huc-exo^TIMP2^ accelerated HUVEC proliferation (Figure 5(c)), migration (Figure 5(d)), and the numbers and length of cell tubes formed *in vitro* (Figure 5(e)) compared to the other treatment groups. Therefore, huc-exo^TIMP2^ promoted angiogenesis in the injured myocardium by promoting endothelial cell proliferation and migration.

### 3.6. huc-exo^TIMP2^ Inhibited Proliferation of CFs and Downregulated MMP2 and MMP9 Expression

**Figure 6:**
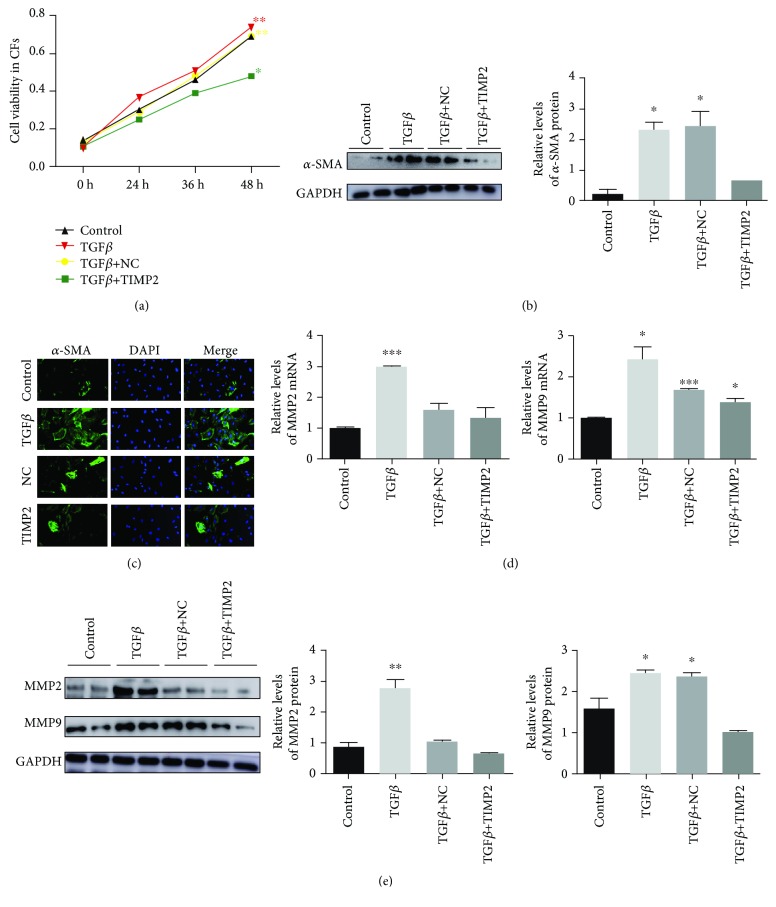
huc-exo^TIMP2^ inhibited cardiac fibroblast proliferation and MMP2/MMP9 production. (a) Cardiac fibroblast proliferation rate measured at 0, 24, 36, and 48 hrs with the CCK8 assay. OD540: optical density at 540 nm. (b) Immunoblots and (c) immunofluorescence showing the expression levels of *α*-SMA in cardiac fibroblasts and comparison of quantified levels. (d, e) Expression levels of MMP2 and MMP9 mRNAs and proteins in the cardiac fibroblasts across different groups. Control: untreated; TGF*β*: treated with TGF*β* alone; NC: treated with huc-exo^NC^ following TGF*β*; TIMP2: treated with huc-exo^TIMP2^ following TGF*β*. ^∗^*p* < 0.05, ^∗∗^*p* < 0.01, and ^∗∗∗^*p* < 0.001.

The cellular basis of myocardial hypertrophy following ischemic injury is the excessive proliferation of CFs. As shown in Figure 6(a), CFs pretreated with huc-exo^TIMP2^ showed significantly lower proliferation rates in a time-dependent manner, in accordance with the protein expression and immunofluorescence level of the myofibroblast marker *α*-SMA which were decreased by huc-exo^TIMP2^ pretreatment (Figures 6(b) and 6(c)). During chronic heart failure, the MMP family of extracellular proteins secreted by the CFs drive ECM remodeling [26] by breaking down the collagen [27]. Consistent with the results so far, huc-exo^TIMP2^ significantly downregulated the levels of MMP2 and MMP9 mRNAs (Figure 6(d)) as well as proteins (Figure 6(e)). Taken together, huc-exo^TIMP2^ alleviated postischemic myocardial remodeling by blocking CF proliferation and secretion.

### 3.7. Regulation of Akt Signaling in MI-Induced Myocardial Apoptosis

**Figure 7:**
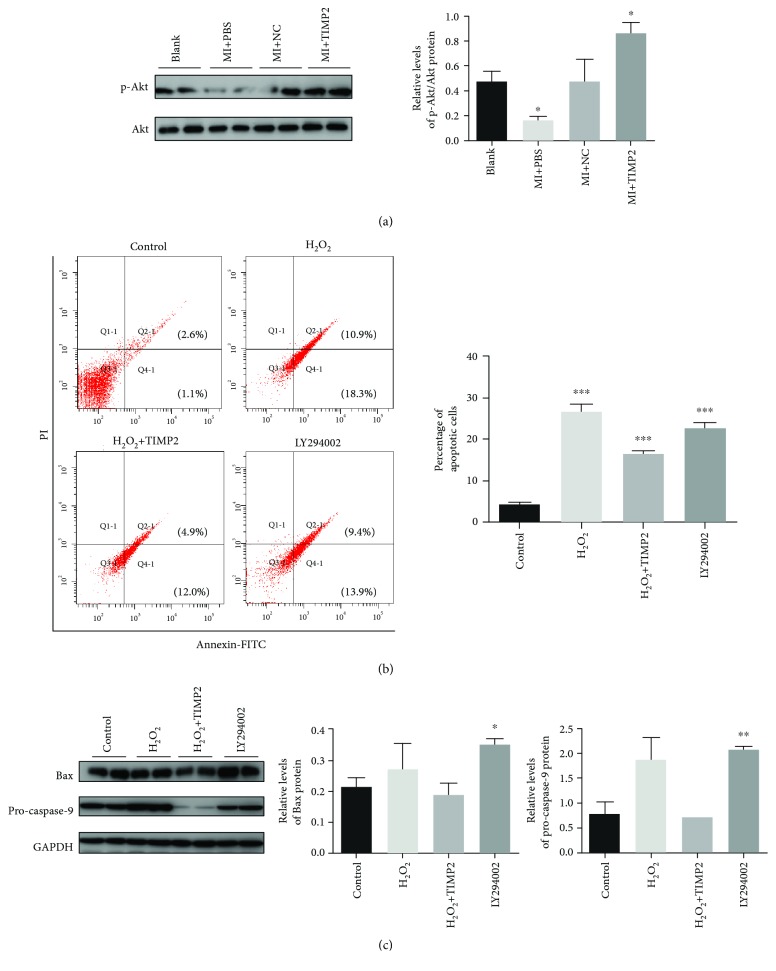
huc-exo^TIMP2^ protects the cardiomyocyte against apoptosis induced by MI injury via the Akt pathway. (a) Immunoblots showing the expression level of phosphorylated Akt in the infarcted rat hearts and quantitative comparison of p-Akt levels across different groups. Blank: rats without surgery; MI+PBS: MI rats treated with PBS; NC: MI rats treated with huc-exo^NC^; TIMP2: MI rats treated with huc-exo^TIMP2^. (b) Flow cytometry plots showing annexin V/PI-stained cells; comparison of the percentage of apoptotic cells across different groups. (c) Immunoblots showing expression levels of Bax and pro-caspase-9 and comparison of protein expression across different groups. Control: untreated; H_2_O_2_: treated with H_2_O_2_ alone; TIMP2: treated with huc-exo^TIMP2^ following H_2_O_2_; LY: treated with LY294002 and huc-exo^TIMP2^ following H_2_O_2_. ^∗^*p* < 0.05, ^∗∗^*p* < 0.01, and ^∗∗∗^*p* < 0.001.

Previous studies have shown that TIMP2 interacted with MT1-MMP complexes, activated the Akt pathway, and inhibited tumor cell apoptosis [21]. Therefore, we hypothesized that huc-exo^TIMP2^ protected the myocardium from apoptosis via the Akt pathway as well. As shown in Figure 7(a), huc-exo^TIMP2^ preconditioning significantly increased the levels of phosphorylated Akt in the injured myocardium. To further validate the mechanistic role of Akt in postischemic apoptosis, we treated cardiomyocytes exposed to H_2_O_2_, an *in vitro* model simulating MI injury, with the Akt inhibitor LY294002. H_2_O_2_ markedly increased the percentage of apoptotic cells to 29%, which was decreased significantly by huc-exo^TIMP2^ treatment to 16.9%. However, in the presence of the Akt inhibitor, the apoptosis rate was high at 23.3% (Figure 7(b)). Consistent with this, LY294002 pretreatment also increased the levels of Bax and pro-caspase-9 in H9C2 cells (Figure 7(c)). Taken together, huc-exo^TIMP2^ protects H9C2 cardiomyocytes from H_2_O_2_-induced apoptosis partly by activating the Akt signaling pathway.

### 3.8. Akt Pathway Is Upstream of Sfrp2 in MI Injury-Induced Response

**Figure 8:**
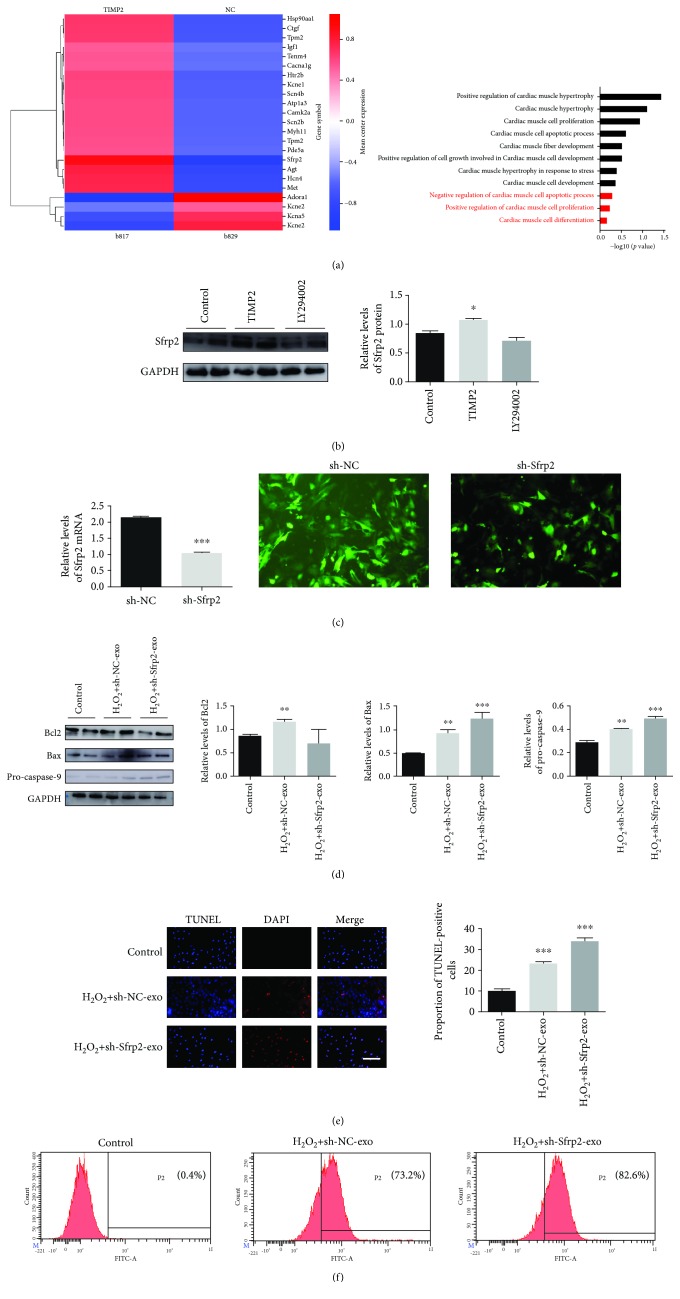
The Akt/Sfrp2 pathway mediates the cardioprotective effects of huc-exo^TIMP2^. (a) Transcriptome sequencing in huc-exo^TIMP2^ and huc-exo^NC^. (b) Immunoblots showing the expression level of Sfrp2 in H9C2 cells; quantitative comparison of the protein levels. Control: untreated; TIMP2: treated with huc-exo^TIMP2^; LY: treated with LY294002 and huc-exo^TIMP2^. (c) RT-PCR and GFP-fluorescent images presenting the transfection efficiency of shRNA-Sfrp2 in hucMSCs^TIMP2^. (d) Immunoblots showing the expression levels of Bcl2, Bax, and pro-caspase-9 in H9C2 cells under H_2_O_2_ stimulation; quantitative comparison of the protein levels. (e) Representative fluorescent images of TUNEL+ in H9C2 cells with the sh-Sfrp2-exo pretreatment. TUNEL (red) and DAPI (blue). Scale bar: 50 *μ*m. Control: untreated. (f) The quantitative analysis of the ROS level evaluated by flow cytometry using DCFH-DA.sh-NC-exo, treated with sh-NC-exo following H_2_O_2_, and sh-Sfrp2-exo, treated with sh-Sfrp2-exo following H_2_O_2_. ^∗^*p* < 0.05, ^∗∗^*p* < 0.01, and ^∗∗∗^*p* < 0.001.

Sfrp2 is an important mediator of the Akt/mTOR pathway during doxorubicin-induced oxidative stress and apoptosis [28]. Transcriptome sequencing showed a significantly higher Sfrp2 expression level in the huc-exo^TIMP2^ compared to the huc-exo^NC^ (Figure 8(a)). Furthermore, LY294002 blocked the huc-exo^TIMP2^-mediated increase of Sfrp2 in H9C2 cardiomyocytes (Figure 8(b)), indicating the involvement of the Akt/Sfrp2 axis in the cardioprotective effects of huc-exo^TIMP2^. To confirm this hypothesis, we knocked down Sfrp2 in hucMSCs^TIMP2^ (sh-Sfrp2) and extracted exosomes (sh-Sfrp2-exo) from them, while exosomes secreted by shRNA-NC-hucMSCs^TIMP2^ were used as a negative control (sh-NC-exo). The RT-PCR and fluorescence GFP results confirmed that Sfrp2 was successfully knocked down in the hucMSCs^TIMP2^ (Figure 8(c)). The Western blot results found a significant increase in the levels of the proapoptotic proteins Bax and pro-caspase-9 and a concomitant decrease in the antiapoptotic protein Bcl2 (Figure 8(d)) in sh-Sfrp2-exo-induced H9C2 cells under H_2_O_2_ stimulation, in addition to a substantial increase in the percentage of TUNEL+ apoptotic cells (Figure 8(e)). Moreover, we confirmed the effect of sh-Sfrp2-exo on H_2_O_2_-induced ROS release. DCFH-DA-stained flow cytometry showed that sh-NC-exo reduced the H_2_O_2_-induced ROS production in H9C2 cells, while sh-Sfrp2-exo remarkably enhanced ROS levels (Figure 8(f)). Taken together, huc-exo^TIMP2^ inhibited apoptosis in the H9C2 cardiomyocytes partly by upregulating Sfrp2.

## 4. Discussion

Stem cell-based therapies have attracted considerable attention in recent years for various degenerative disorders, and encouraging results have been observed in animal studies and clinical trials. However, large-scale clinical application of regenerative therapies is limited due to the low cellular survival rate in the ischemic microenvironment [29], primarily due to the high levels of ROS that can cause oxidative damage and reduce the viability of the transplanted stem cells [30]. A recent study has demonstrated that human amniotic fluid-derived MSC-utilized hypoxic conditioned media have more paracrine factors, VEGF and TGF*β*1, and secretes and enhances the proliferation and migration of human dermal fibroblasts *in vitro* and wound closure in a skin injury model, as compared to normoxic conditioned media [31]. It is well thought that stem cells exert therapeutic effects by releasing extracellular vesicles or trophic factors [32]. Indeed, cell-to-cell interaction is orchestrated by extracellular vesicles, which are involved in cell communication by transferring protein, mRNA, and lipids to target cells.

Exosomes, a kind of extracellular vesicle, are released from and taken up by different cell types [33, 34]. For example, exosomes released from stem cells have been shown to ameliorate ischemia injury [35] and prevent adverse remodeling of the myocardium [36]. In our work, we successfully exploited lentivirus transfection TIMP2-modified hucMSCs and collected their exosomes. We subsequently demonstrated the exosome's shape and detected the expression of exosomal markers CD9 and CD63. Importantly, our results suggested that TIMP2-modified hucMSC-derived exosomes ameliorate MI injury, reducing myocardial apoptosis and fibrosis, and improve cardiac function by attenuating oxidative stress and ECM remodeling. Mechanistically, we have investigated the antiapoptotic function and anticardiac fibroblast proliferation, further demonstrating that the effects resulted, at least in part, from activating the Akt/Sfrp2 pathway.

Previous works have showed that oxidative stress in the injured myocardium results in cardiomyocyte apoptosis, which persists throughout the cardiac remodeling and significantly influences the outcome of anti-ischemia therapy [37]. Therefore, blocking apoptosis in the cardiomyocytes can also alleviate cardiac dysfunction and inhibit the development of myocardial remodeling [38, 39]. Since the MMP2 inhibitor TIMP2 blocked oxidative stress-induced apoptosis in a mouse model of ischemic stroke [25], and showed high endogenous levels in primary hucMSCs in our work, we hypothesized its cardioprotective effect in MI injury. We therefore isolated exosomes secreted by TIMP2-overexpressing hucMSCs in order to determine their potential therapeutic effects in a rat model of MI.

The antiapoptotic action of huc-exo^TIMP2^ in the cardiomyocytes was evaluated in terms of the significantly reduced proportion of TUNEL+ cells, as well as decreased the Bax/Bcl2 ratio and pro-caspase-9 levels. The balance between Bax and Bcl2 is responsible for the integrity of the mitochondrial membrane, and any disruption in this balance results in mitochondrial depolarization and the subsequent activation of the apoptotic cascade [40]. In addition, excessive production of ROS overwhelms the endogenous antioxidant defense system, resulting in massive cellular damage through peroxidation of membrane lipids, sulphydryl enzyme inactivation, and DNA breakdown, which triggers apoptotic cell death [41]. We found low antioxidant enzyme activity in the infarcted rat hearts, which was reversed by huc-exo^TIMP2^ treatment. Thus, TIMP2 restores the endogenous antioxidant defense system in the myocardium following ischemic injury.

ECM remodeling primarily involves increased MMP9 activity and is accompanied by decreased TIMP2 levels. MMP9 results in excessive collagen deposition and fibrosis in the ECM by inducing antiangiogenic factors in the hypertrophied myocardium [42]. TIMP2 is highly expressed in the myocardium and has the dual actions of activating pro-MMP2 and blocking the activation of MMP2, which inhibit angiogenesis and the development of heart failure [43]. Interestingly, excess TIMP2 also inhibits MMP2 expression [44]. In addition, MMP9 lead to collagen deposition and fibrosis by inducing antiangiogenic factors in the hypertrophied myocardium [42]. In the aortic banding mouse model, TIMP2 knockout resulted in very poor left ventricular function and significant myocardial fibrosis [45]. Consistent with these findings, replenishing TIMP2 in the diseased myocardium proved therapeutic by reducing or preventing disease progression [18]. Furthermore, ROS also activate MMP enzymes and inactivate their endogenous inhibitors [46], which disrupt the balance between matrix deposition and degradation, ultimately leading to myocardial remodeling and impaired cardiac function. In line with all the previous studies, our data suggest that huc-exo^TIMP2^ decrease the levels of both MMP2 and MMP9 and improved heart function following MI injury with FS% increase, which is similar in magnitude to the increase in LVEF%. Recent clinical trials reported that this improvement in FS% translated to enhanced ventricular function, end-systolic elastance, and preload recruitable stroke work [47, 48].

Angiogenesis, which is vital for repairing the ischemic microenvironment, depends on the migration and proliferation of vascular endothelial cells. Zhang et al. showed that hucMSC-derived exosomes accelerated angiogenesis via the Wnt4/b/catenin signaling pathway [49]. Bian et al. reported that vesicles secreted by the human bone marrow mesenchymal stem cells increased angiogenesis in a rat model of MI [50]. In the present study, we demonstrated that huc-exo^TIMP2^ increased the number of CD31- and lectin-immunoreactive cells in the MI rat myocardium. It also promoted the proliferation, migration, and tube forming ability of HUVECs under H_2_O_2_ exposure *in vitro*. These therapeutic effects might be through the delivery of signaling proteins, enzymes, and transcription factors by exosomes, which could regulate the signaling and function in recipient cells. We found that Akt was significantly higher in huc-exo^TIMP2^ transplanted myocardial tissue compared with huc-exo^NC^, indicating that the huc-exo^TIMP2^ might have delivered some factors to the recipient cells and exerted signal communication.

The Akt pathway is activated by ischemic injury and is pivotal for regulating cardiomyocyte survival [51]. Kim et al. reported that TIMP2 stimulated lung adenocarcinoma cell proliferation by activating the PI3K/Akt pathway activation in an MMP-independent manner [52]. Gnecchi et al. found that Akt-overexpressing MSCs reduced infarct size and restored cardiac function in mice hearts after MI; they postulated that Sfrp2 is a key factor in the protective effect [53]. Sfrp2 was first shown as a downstream target of the PI3K/Akt pathway by Gehmert et al. [54]. Alfaro et al. [55] also found that Sfrp2 inactivated the caspase network and promoted MSC survival. Based on these reports, we determined whether activated Akt induced by huc-exo^TIMP2^ could upregulate Sfrp2 and whether the latter was involved in maintaining the balance of pro- and antiapoptotic factors. We found that huc-exo^TIMP2^ could upregulate the Sfrp2 level in H9C2 cardiomyocytes, and the expression of the latter was Akt dependent. In addition, Sfrp2 knockdown in huc-exo^TIMP2^ increased the Bax/Bcl-2 ratio and pro-caspase-9 levels. Taken together, Akt is upstream of Sfrp2 during the oxidative stress-induced response, thus validating the essential prosurvival role of Sfrp2 [56]. However, the underlying molecular mechanisms need further investigation.

## 5. Conclusion

In conclusion, exosomes derived from TIMP2-modified hucMSCs repaired the ischemic myocardium by inhibiting cardiomyocyte apoptosis and promoting angiogenesis and ECM remodeling, partly by activating the prosurvival Akt/Sfrp2 pathway. This study establishes a new promising therapeutic strategy against MI, which needs further clinical validation.

## Data Availability

The data used to support the findings of this study are included within the article.
